# Editorial: Nanotechnology and smart textiles: Sustainable developments of applications

**DOI:** 10.3389/fbioe.2022.1002887

**Published:** 2022-08-26

**Authors:** Amr Fouda, Mohammed F. Hamza, Th. I. Shaheen, Yuezhou Wei

**Affiliations:** ^1^ Department of Botany and Microbiology, Faculty of Science, Al-Azhar University, Cairo, Egypt; ^2^ School of Nuclear Science and Technology, University of South China, Hengyang, China; ^3^ Nuclear Materials Authority, Cairo, Egypt; ^4^ National Research Centre, Textile Research Division, Cairo, Egypt; ^5^ School of Nuclear Science and Engineering, Shanghai Jiao Tong University, Shanghai, China

**Keywords:** smart textiles, nanomaterials, UV-protection, antibacterial activity, green synthesis

The huge development in nanotechnology has greatly introduced prospective opportunities to construct improved materials with sophisticated properties to be incorporated into various fields. Such applications are due to the unique properties of nanomaterials originating from the nanoscale structure which are completely different from its bulk materials. One of the current challenges is the possible spread of viral and bacterial infections in the healthcare units, small clinics, doctor’s offices, and hospitals that threaten the safety of the healthcare team, visitors, and patients. Textiles and clothes are one of the reasons for spreading infections between patients and others as they are in direct contact with the skin, therefore can be named “second skin”. Therefore, it is urgent to improve the overall properties of textile used for medicinal applications. Nanotechnology can contribute to producing smart textiles with multi-functional properties such as UV protection, flame-retardant, oil, and water repellent, antimicrobial, wrinkle resistance, anti-odor, and antistatic properties. Various chemically and physically-generated metal and metal oxide nanoparticles are applied for smart textiles. However, nanomaterials produced by green approaches are preferred to eliminate or minimize the harmful effects of traditional methods.

This issue contains seven articles, two review articles, and five original research papers, covering the current research topic. The first research article (Sadeghi-Kiakhani et al.) focused on the enhancement of the antibacterial and antioxidant properties of cotton fabrics using silver nanoparticles (Ag-NPs). Whereas, the following three original research articles (Fouda et al.; Giaconica et al.; Lotfy et al.) focused on the fabrication of nanofiber, magnesium oxide nanoparticles (MgO-NPs), and silver nanoparticles (Ag-NPs) by green approaches. The last research article (Guo et al.) was concerned with the formation of biochar to remove radioactive strontium from artificial seawater. The two review articles (Hu et al. and Pan et al.) highlighted the utilization of metal and metal oxide nanomaterials to reduce metabolic disorders as well as therapeutic applications.

An improvement of antibacterial and antioxidant activities of cotton fabrics by incorporating nanomaterials was investigated by Sadeghi-Kiakhani et al. In this study, the cotton fabrics were dyed with natural colorant extracted from dried petals of *Malva sylvestris* in the presence of tannic acid as a bio-mordent. The natural dye loaded on cotton fabrics was used as a biocatalyst to reduce AgNO_3_ to Ag-NPs with sizes in ranges of 50–80 nm. The obtained cotton fabrics showed the highest growth reduction of *Staphylococcus aureus* and *Escherichia coli* with values of 99%, this value was decreased with percentages of 3% after ten washing cycles. The authors concluded that the properties of cotton fabrics such as washing and light fastness were enhanced from moderate to good and then to very good after adding the mordant.

Fouda et al. have successfully fabricated MgO-NPs through harnessing metabolites of brown macroalgae *Cystoseira crinita* as a green approach. The authors have characterized the biosynthesized MgO-NPs using UV-vis spectroscopy (UV-Vis), Transmission Electron Microscopy (TEM), Fourier transforms infrared (FT-IR), Energy-dispersive X-ray (SEM-EDX), X-ray photoelectron spectroscopy (XPS), and X-ray diffraction (XRD). They reported that the bio-fabricated MgO-NPs exhibited promising antimicrobial, *in-vitro* cytotoxicity, mosquitocidal, and repellence activities in a dose-dependent manner. Their data analysis revealed that the minimum inhibitory concentration (MIC) against *Staphylococcus aureus, Bacillus subtilis, Pseudomonas aeruginosa, Escherichia coli,* and *Candida albicans* was at low NPs concentrations (12.5–50 μg mL^−1^)*.* Also, the synthesized MgO-NPs showed target orientation to Caco-2 cancer cell lines at low concentrations as compared to Vero normal cell lines. Moreover, the MgO-NPs at 10 μg mL^−1^ exhibited the highest mortality percentages against various instar larvae (I, II, III), and pupa of *Musca domestica* were 99%, 95%, 92.2%, and 81%, respectively.

Interestingly, Giaconia et al. studied the assembly of nanofibers (NFs) from fermented jussara pulp (FJP) and polyethylene oxide (PEO) using the electrospinning technique and evaluated their characteristics. Additionally, they determined the antioxidant activity (AA) and bioaccessibility of the FJP and its polymeric solution with PEO, a step that precedes the NFs fabrication, to access the protective role of the polymer on the bioactive compounds present in the pulp. Data showed that the nanofibers were successfully formed with a diameter size of 101.2 ± 26.2 nm. Besides, the authors have determined the FE-SEM analysis, thermal gravimetric analysis (TGA), FTIR, and hydrophobicity of fermented jussara NFs. The FJP NF synthetized thermal stability was evaluated using TG, and the thermal gravimetric analysis (TGA) derived thermogravimetry (DTG) curves for fermented jussara NFs.

The role of mycelial metabolites of *Aspergillus terreus* strain BA6 to fabricate Ag-NPs was assessed by Lotfy et al. The activities of NPs were size-dependent, therefore, the authors investigated the effect of seven independent variables [pH values for media and reactions, inoculum size (spores/mL), metal precursors (AgNO_3_) concentrations (mM), peptone and dextrose concentrations (g/L), and the ratio of metal precursor to mycelial filtrate (*v/v*)] on the size of the synthesized Ag-NPs. Under the optimal conditions, spherical, well dispersed, and crystallographic Ag-NPs were formed with sizes of 7–23 nm. The final product exhibited high antibacterial and antifungal activities against various pathogenic strains with MIC values in ranges of 0.31–1.25 μg mL^−1^ and MBC/MFC values in ranges of 0.62–10 μg mL^−1^. Also, the synthesized Ag-NPs exhibited cytotoxic efficacy against breast cancer cells (Mcf-7) with an IC_50_ value of 87.5 μg mL^−1^.

An interesting research article by Guo et al. used a teak peel modified biochar (PMBN3) as a cost-effective and environmentally friendly material for radioactive water treatment and useful for environmental remediation. In this study, CO_2_ nanobubble co-precipitation was used to remove the majority of Sr(II) from artificial seawater, and the remaining Sr(II) in the solution was adsorbed by an as-prepared biochar adsorbent. The removal of more than 99% of Sr(II) ion was performed after precipitation of the interfering metal ions using CO_2_ nanobubbles. The prepared material was performed through rapid pyrolysis and oxidative modification to remove Sr(II) from artificial seawater. The material was characterized by rich functional groups (oxygen-containing groups, i.e. carboxyl and hydroxyl groups) that were used for rapid removal of Sr ions from the solution, laminar morphology, and large specific surface area (characterized by mesoporous structure). The batch method was used to investigate the sorption characterization and the sorbent was characterized by fast adsorption kinetics (less than 1 h for equilibrium, with PSORE and internal diffusion kinetic models), the Freundlich model is the most equation for fitting the sorption isotherm (*R* ^2^ > 0.98) and superior reusability properties.

The review article by Hu et al. focused on the efficacy of nanostructure fabricated by plant extracts in the management and control of metabolic disorders such as insulin resistance, systematic hypertension, obesity, and atherogenic dyslipidemia, which cause metabolic syndrome. The authors highlighted several delivery systems based on nanostructures such as nanocomposites hydrogel, solid lipid, liposomes, nano-emulsions, core-shell NPs, and micelles especially those synthesized by plant extracts. These systems were concluded as promising tools for decreasing (*in-vitro* and *in-vivo*) oxidative stress, chronic inflammation, lipid profile, and insulin resistance through interference with metabolic syndrome pathways, and hence profitable in clinical applications. On the other hand, the mini-review by Pan et al. demonstrates the recent advancements in the applications of metal and metal oxide-NPs in cosmetic, skin, and wound healing. The authors highlighted the integration of synthesized NPs in dermato-cosmetic applications such as drug releases that have dual properties to protect the skin from UV- and bacterial infections. Moreover, the authors discussed one important skin disease namely psoriasis which can infect any human skin part and appear as red (erythematous) or flaking. Finally, they discussed the role of different NPs such as Au and Ag in the treatment of this chronic skin disease.

In conclusion, the articles published in the current research topic highlighted the role of different nanomaterials in the control of pathogenic microbes, wound healing, UV protection, metabolic disorders, skin and cosmetic therapeutics, and their role in the improvement the textile fabrics to produce smart textiles with unique properties.

